# The Role of Fragile Sites in Sporadic Papillary Thyroid Carcinoma

**DOI:** 10.1155/2012/927683

**Published:** 2012-06-11

**Authors:** Laura W. Dillon, Christine E. Lehman, Yuh-Hwa Wang

**Affiliations:** ^1^Department of Biochemistry, Wake Forest School of Medicine, Medical Center Boulevard, Winston-Salem, NC 27157-1016, USA; ^2^Department of Cancer Biology, Wake Forest School of Medicine, Medical Center Boulevard, Winston-Salem, NC 27157-1016, USA

## Abstract

The incidence of thyroid cancer is increasing, especially papillary thyroid carcinoma (PTC), making it currently the fastest-growing cancer among women. Reasons for this increase remain unclear, but several risk factors including radiation exposure and improved detection techniques have been suggested. Recently, the induction of chromosomal fragile site breakage was found to result in the formation of *RET/PTC1* rearrangements, a common cause of PTC. Chromosomal fragile sites are regions of the genome with a high susceptibility to forming DNA breaks and are often associated with cancer. Exposure to a variety of external agents can induce fragile site breakage, which may account for some of the observed increase in PTC. This paper discusses the role of fragile site breakage in PTC development, external fragile site-inducing agents that may be potential risk factors for PTC, and how these factors are especially targeting women.

## 1. Introduction

The incidence of thyroid cancer is dramatically rising in the Unites States and other countries. Thyroid cancer has increased steadily in the Unites States over the past several decades, and according to data from the National Cancer Institute's Surveillance, Epidemiology and End Results (SEER) database, incidences are now nearly three times that of the early 1970s [1–3]. Furthermore, for unknown reasons, thyroid cancer is three times more prevalent in women than men. Thyroid cancer is the sixth most common type of cancer among women and increasing more rapidly than any other cancer. The American Cancer Society estimates that 56,460 new cases of thyroid cancer (43,210 in women and 13,250 in men) will be diagnosed in the United States in 2012, with approximately 80% of patients below 65 years old [4].

The recent upsurge in thyroid cancer is not well understood. Rates of thyroid cancer diagnoses have increased the most among small (≤2 cm) thyroid nodules, which may be explained by the use of thyroid ultrasound for diagnosis beginning in the 1980s [2, 5]. However, it is believed that the increase in thyroid cancer is not solely based on diagnostic methods [6], but due to changes in other risk factors as well [7]. One possible contributory risk factor is exposure to ionizing radiation [8], either from external radiation, such as X-rays and *γ*-radiation, or internal radiation, from ingestion or inhalation of radioiodine. Increases in thyroid cancer have been well documented following exposure to high doses of radiation during medical procedures or following nuclear bomb explosions or nuclear reactor fallouts [9]. However, exposure to low doses of radiation from routine diagnostic X-ray procedures and in the workplace does not increase the risk of thyroid cancer development [9], suggesting radiation exposure is not the only risk factor. An increased body mass index (BMI) is also positively associated with thyroid cancer in both women and men [10], suggesting obesity is another risk factor for thyroid cancer.

Recently, it was observed that the chemical induction of DNA breakage at chromosomal fragile sites could result in the formation of *RET/PTC1* rearrangements, one common mutation observed in papillary thyroid carcinoma (PTC) [11]. Chromosomal fragile sites are regions of the genome prone to DNA breakage and often coincide with mutations observed in cancer [12]. Exposure to a variety of external agents, including dietary, environmental, and chemotherapeutic agents, can induce breakage at fragile sites. Therefore, exposure to fragile-site-inducing conditions may be an additional risk factor for thyroid cancer development, specifically PTC. Interestingly, the increasing rates of thyroid cancer are almost entirely due to an increase in PTC [2, 3, 13]. In this paper, we will discuss the potential role of fragile sites in PTC and the external fragile-site-inducing agents that may be a risk factor for thyroid cancer.

## 2. Fragile Sites and Cancer

### 2.1. Chromosomal Fragile Sites

Chromosomal fragile sites are nonrandom loci that can be observed as gaps or breaks on metaphase chromosomes under conditions that partially inhibit DNA replication [14]. Fragile sites can further be defined as common or rare, based on the frequency of their occurrence in the population. Rare fragile sites consist of repeated sequence motifs, such as trinucleotide repeats, which are present in less than 5% of the population and are inherited in a Mendelian manner [15]. In contrast, common fragile sites are present in all individuals and therefore are a normal component of chromosomal architecture [16]. 

Fragile sites can be observed in culture through treatment with various chemicals. The majority of common fragile sites can be induced by aphidicolin (APH), an inhibitor of DNA polymerases *α*, *β*, and *δ* [17, 18]. Induction of other common fragile sites has been observed following treatment with bromodeoxyuridine (BrdU) or 5-azacytidine [19]. Most rare fragile sites are expressed through the removal of folate, while others show induction following treatment with distamycin-A or BrdU [15]. Additionally, common fragile site breakage can be induced or enhanced through exposure to various dietary or environmental chemicals, including chemotherapeutic agents [12] (details in Section 4).

Unlike rare fragile sites, no known consensus sequence exists for common fragile sites. However, several characteristics have been observed at many common fragile sites studied to date. These include being located within large genes [19] and within regions of the genome that replicate late in the S-phase [20–23]. Also, several fragile sites contain highly flexible AT-rich sequences [24, 25] and are predicted to form stable DNA secondary structures [24, 26, 27]. Also, the ATR (ataxia-telangiectasia and Rad3-Related-) dependent DNA repair pathway, which responds to stalled or collapsed replication forks, is known to be vital for maintaining stability at fragile sites [28–30]. One model for common fragile site breakage is that, under conditions of replication stress, stable DNA secondary structures form at fragile sites, blocking replication fork progression. If the ATR pathway fails to properly repair these stalled replication forks, this could result in DNA breakage within these regions. In addition to replication fork stalling, paucity of replication initiation in fragile site regions [31] and the presence of transcription-derived R-loops during DNA replication of fragile sites [32] may also be involved in the mechanism of fragility.

### 2.2. Role of Fragile Sites in Cancer

Studies over the past several decades have shown a correlation between fragile sites and cancer-specific chromosomal aberrations [33]. Many of the genes identified within fragile sites are known tumor suppressor genes or oncogenes [34]. Fragile sites have been identified as hot spots for sister chromatid exchange [35], viral integrations [36–41], and gene amplifications [42–45] in tumor cells. Additionally, mutational signatures of some unexplained homozygous deletions observed in cancer cell lines match those at fragile sites [46].

**Figure 1:**
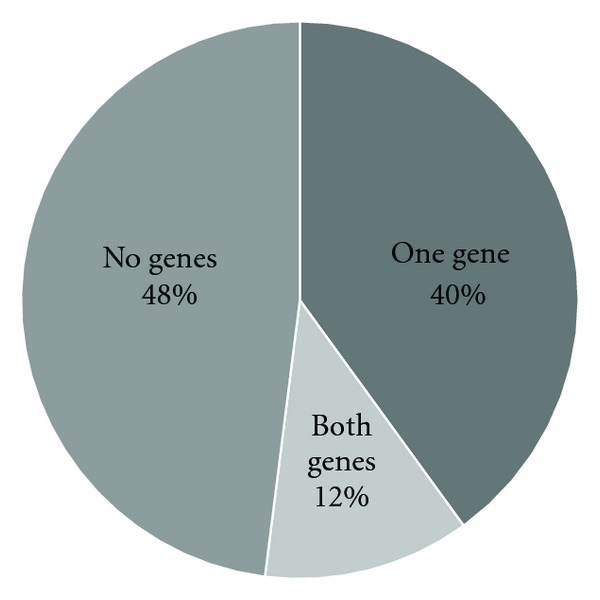
Percentage of breakpoints in genes involved in cancer-causing simple recurrent chromosomal translocations located within fragile sites.

A comprehensive examination of all known simple chromosomal translocations in cancer revealed that 52% of these recurrent translocations had at least one breakpoint located within a fragile site [47]. Specifically, 40% of translocations had breakpoints within one gene located in a fragile site, while an additional 12% of translocations had breakpoints within both genes located in fragile sites (Figure 1). Furthermore, 65% of the breakpoints identified within fragile sites were within common—not rare—fragile sites, conferring a genetic risk among all individuals. Since this study only focused on simple translocations between two genes and not the participation of fragile sites in other more complex genomic rearrangements, the association of fragile sites with breakpoints in cancer may prove to be greater than estimated in this study.

In addition to the correlation between fragile sites and regions of the genome mutated in cancer, two studies have investigated the direct contribution of fragile site breakage to the formation of cancer-specific chromosomal aberrations. Durkin et al. observed deletions within the tumor suppressor gene *FHIT*, located within the most active common fragile site FRA3B, following treatment of human-mouse chromosome 3 somatic hybrid cells with APH. These deletions were consistent with those observed in esophageal, breast, and lung cancers [48]. Recently, we observed the formation of *RET/PTC1* rearrangements, a translocation commonly observed in PTC, in a human thyroid epithelial cell line following treatment with the fragile-site-inducing chemicals APH, BrdU, and 2-aminopurine (2-AP) [11].

Individuals genetically predisposed to forming cancer also have higher levels of fragile site breakage. For example, Seckel syndrome—a rare genetic disorder in which patients exhibit high levels of chromosomal instability and cancer—is caused by low expression of the DNA repair protein ATR, due to a hypomorphic mutation in the *ATR* gene [49]. Cells from patients with Seckel syndrome have significantly higher levels of APH-induced fragile site breakage compared to normal individuals [50]. Another rare genetic disorder, Fanconi anemia (FA), is the result of mutations in various proteins involved in the FA double-strand DNA repair pathway. Patients with FA have elevated levels of chromosomal breakage and cancer [51]. Chromosomal breakpoints in blood lymphocytes from FA patients are preferentially located in fragile sites [52], and APH-induced fragile site breakage is significantly increased among these patients [53]. Proteins in both the ATR and FA DNA repair pathways are important in maintaining stability at fragile sites [12]. The WRN protein, which is phosphorylated by and colocalizes with ATR in response to replication fork arrest [54], is also vital for maintaining common fragile site stability, and this function requires WRN helicase activity [55]. Mutations in WRN can result in Werner syndrome, which is an autosomal recessive premature aging disease where individuals have a high predisposition to cancer development [56]. Interestingly, Japanese Werner syndrome patients have much higher levels of thyroid cancer, including PTC, than normal Japanese individuals [57]. Together these provide extreme examples for a genetic predisposition for fragile site breakage and cancer development.

Together these previous studies provide a strong link between fragile sites and cancer, whereby exposure to fragile-site-inducing conditions and/or a genetic predisposition could attribute to the development of various types of cancer.

## 3. Fragile Site Instability in PTC

### 3.1. RET/PTC Translocations in PTC

PTC is primarily responsible for the upsurge in thyroid cancer rates [3]. One mutation commonly observed in PTC is *RET/PTC* rearrangements, in which the *RET* oncogene translocates with a variety of genes that are constitutively expressed in the thyroid. *RET* (rearranged during transfection) encodes for a cell membrane receptor tyrosine kinase that responds to ligands of the glial cell line-neurotropic factor (GDNF) family, activating cell growth and survival pathways [58]. Expression of *RET* in the thyroid is high in neural-crest-derived C cells but not in follicular cells, where *RET/PTC* rearrangements result in its activation through expression of fusion proteins leading to tumorigenesis. 

While prevalence of *RET/PTC* rearrangements is variable among different studies, overall these translocations are found in 30–40% of adult and 50–60% of pediatric PTC tumors [59]. To date, 12 *RET/PTC* rearrangements have been reported, all involving *RET* [60]. The two most common subtypes are *RET/PTC1* and *RET/PTC3*, where *RET* is translocated with *CCDC6 *and *NCOA4*, respectively [61]. One known risk factor for *RET/PTC* rearrangement formation is exposure to radiation, where the incidence of *RET/PTC* rearrangements in PTC patients increases to 60–70% regardless of age [59]. *RET/PTC3* rearrangements have shown a strong correlation with radiation exposure, where multiple studies indicated these translocations in 63–75% of radiation-induced *RET/PTC*-positive pediatric PTC tumors [62–65]. In contrast, *RET/PTC1* rearrangements have been observed in 50–71% of *RET/PTC*-positive sporadic PTC tumors, while *RET/PTC3* rearrangements were only observed in 13–42% of tumors [62, 66–68].

Spatial proximity of *RET/PTC*-participating genes is one of the factors contributing to cell specificity of the disease [69]. The interphase distance between *RET* and *CCDC6* is shorter in normal human thyroid cells than in peripheral blood lymphocytes or in normal mammary epithelial cells [69]. Further, *RET*, *CCDC6*, and *NCOA4* are all located on chromosome 10 and despite the predicted distance of the genes based on their location along chromosome 10, *RET* is located closer to *NCOA4* and *CCDC6* than would be expected, during interphase in normal human thyroid cells [70].

*RET*, *CCDC6*, and *NCOA4*, the genes participating in *RET/PTC1* and *RET/PTC3* rearrangements, are all located within common fragile sites. *RET* and *NCOA4* are both located within the same APH-inducible common fragile site, FRA10G, while *CCCD6* is located within the BrdU-induced common fragile site FRA10C. The location of these genes within fragile sites, the unexplained nature of sporadic PTC tumors containing translocations of these genes, and the increasing incidence of PTC tumors suggest that fragile site breakage may contribute to sporadic *RET/PTC* rearrangements. 

### 3.2. Fragile-Site-Inducing Conditions Produce DNA Breaks in RET/PTC Rearrangement-Participating Genes and Generate RET/PTC1 Translocations

**Figure 2:**
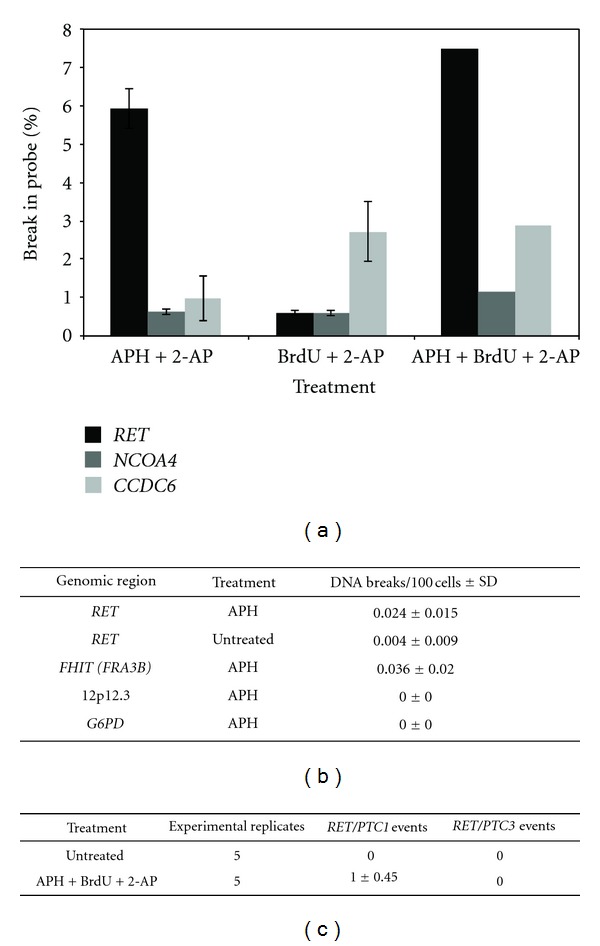
Fragile-site-inducing chemicals generate DNA breakage within *RET/PTC* genes and *RET/PTC1* rearrangements. (a) Percentage of chromosomes showing disruption of *RET*, *NCOA4*, and *CCDC6* following treatment of HTori-3 cells with fragile-site-inducing chemicals as detected by FISH. Error bars represent standard deviation. (b) The level of DNA breakage in HTori-3 cells at *RET* intron 11, *FHIT* intron 4, 12p12.3, and *G6PD* with or without APH treatment was detected using LM-PCR. (c) The formation of *RET/PTC1* or *RET/PTC3* rearrangement events was detected in HTori-3 cells using RT-PCR following treatment with fragile-site-inducing chemicals. Five experimental replicates were performed for each treatment and the average number of rearrangements detected per 10^6^ cells per experiment is shown.

The idea that fragile site breakage contributes to *RET/PTC* rearrangement formation was directly demonstrated in our recent publication [11]. In this study, we first examined whether *RET*, *CCDC6*, and *NCOA4* are true fragile sites, for example, sensitive to fragile-site-inducing conditions using fluorescence *in situ* hybridization (FISH). HTori-3 cells, a human thyroid epithelial cell line devoid of *RET/PTC* rearrangements, were treated with combinations of fragile-site-inducing chemicals known to induce the fragile sites containing the *RET/PTC* genes, and chromosomal breakage was measured at each gene based on the percent break of the corresponding FISH probe (Figure 2(a)). The presence of *RET* and *NCOA4* in the APH-induced common fragile site FRA10G was investigated by treating HTori-3 cells with APH and 2-AP. The chemical 2-AP is a general inhibitor of ATR kinase, shown to increase fragile site breakage at FRA3B [29]. This treatment produced significant levels of chromosomal breakage at *RET* and only low levels at *NCOA4* and *CCDC6*, indicating that *RET* and not *NCOA4* is contained within FRA10G. The presence of *CCDC6* within the BrdU-induced common fragile site FRA10C was tested by treating HTori-3 cells with BrdU and 2-AP. High levels of breakage were observed at *CCDC6*, but only low levels at *RET* and *NCOA4* (Figure 2(a)), confirming the location of *CCDC6* within FRA10C. These observations demonstrated that fragile-site-inducing chemicals consistent with the mode of induction for each fragile site can induce DNA breakage at *RET* and *CCDC6* in thyroid cells. Furthermore, when HTori-3 cells were treated with all three chemicals at once, high levels of breakage were observed within *RET* and *CCDC6* simultaneously (Figure 2(a)), indicating the possibility of *RET/PTC1* chromosomal rearrangements.

The major breakpoint cluster region of *RET* observed in *RET/PTC* tumor cells is intron 11 [71]. Using ligation-mediated-PCR (LM-PCR), we showed that APH treatment induced DNA breakage within intron 11 of *RET* in HTori-3 cells. The rate of DNA breakage at *RET* intron 11 with APH treatment was significantly greater than without treatment (*P* = 0.010, Figure 2(b)). Furthermore, APH-induced breakage in HTori-3 cells was specific to fragile sites, where APH treatment also induced DNA breakage within *FHIT*, located within the APH-inducible common fragile site FRA3B, but not in the nonfragile 12p12.3 region or in the *G6PD* gene, located within the non-APH-inducible rare folate-sensitive fragile site FRAXF (Figure 2(b)). Furthermore, the breakpoints located within intron 11 of *RET* were located near previously identified breakpoints in *RET/PTC* tumors [72, 73]. These results suggest that fragile site breakage within *RET* could lead to the generation of oncogenic fusion transcripts.

The generation of *RET/PTC* rearrangements following exposure to fragile-site-inducing chemicals was directly tested by treating HTori-3 cells with APH, BrdU, and 2-AP, and the presence of fusion *RET/PTC1* or *RET/PTC3* mRNA transcripts was detected by reverse transcription-PCR (RT-PCR). No *RET/PTC* rearrangements were detected without treatment, indicating an extremely low level of spontaneous rearrangement (Figure 2(c)). However, treatment with fragile-site-inducing chemicals resulted in *RET/PTC1* rearrangement events with a frequency of one in 10^6^ cells, but no *RET/PTC3* rearrangement events (Figure 2(c)). These results were consistent with the chromosomal breakage observed in FISH analyses, where only the *RET/PTC1* genes *RET* and *CCDC6* exhibited high levels of breakage following treatment.

The data from this study provide direct evidence that fragile sites are involved in the generation of *RET/PTC1* rearrangements in sporadic PTC tumors. Exposure to external factors that can induce fragile site breakage may play a role in the increasing incidence of PTC.

## 4. Effect of External Factors on Fragile Site Breakage

Aside from classic fragile-site-inducing chemicals like APH, DNA breakage at common fragile sites has been observed following exposure to many external agents, including dietary, environmental, and chemotherapeutic compounds (Table 1). Variability in fragile site breakage has been observed among individuals [87], with high levels being associated with cancer patients [88]. These variances may reflect differing exposures to external fragile-site-inducing agents, and such exposure may predispose an individual to a variety of cancers, including PTC.

### 4.1. Environmental and Dietary Fragile-Site-Inducing/Enhancing Chemicals

Numerous dietary and environmental chemicals can induce or enhance fragile site breakage (Table 1). Caffeine and ethanol are two dietary agents that can significantly increase the rate of fragile site breakage. Caffeine, an inhibitor of phosphoinositide 3-kinase-related kinases, including ATR and ATM (ataxia telangiectasia mutated), significantly enhances fragile site breakage in combination with APH or fluorodeoxyuridine (FUdR) [76, 77]. Similarly, ethanol enhances APH-induced fragile site breakage [80]. Cells from chronic alcohol users have an increased frequency of fragile site breakage compared to nondrinkers, which suggests that long-term alcohol use can induce fragile site expression [81].

Exposure to cigarette smoke, pesticides, or hypoxic conditions can also increase susceptibility to fragile site breakage. Peripheral blood lymphocytes from cigarette smokers have significantly greater levels of APH-induced fragile site breakage compared to nonsmokers [78]. Interestingly, peripheral blood lymphocytes from non-smokers and patients with small cell lung cancer who have stopped smoking both display lower levels of fragile site breakage following APH treatment than active smokers, suggesting this risk factor is reversible [79]. Individuals exposed to pesticides through occupational work, such as pesticide sprayers or flower collectors working in greenhouses, have increased levels of APH-induced fragile site breakage in their blood lymphocytes compared to control individuals, and these results persisted even a year later [83–85]. Furthermore, the pesticide-induced breakage was located within fragile sites containing breakpoints observed in non-Hodgkin's lymphoma and leukemia; consistent with this finding, increasing rates of hematopoietic cancers have been linked to pesticide exposure [89, 90]. Hypoxic conditions also enhance fragile site breakage with or without APH treatment in CMA32 Chinese hamster cells [82].

Dietary and environmental agents, carbon tetrachloride, dimethyl sulfate, benzene, and diethylnitrosamine all can induce fragile site breakage [75]. Carbon tetrachloride is used in refrigerants, pesticides, and industrial and manufacturing processes [91]. Formerly, this compound was used in cleaning fluids and fire extinguishers, but has since been banned from home use due to its carcinogenic properties. An increased risk of non-Hodgkin's lymphoma has been reported among individuals working in manufacturing, industrial, and laboratory jobs in which they are exposed to carbon tetrachloride. Dimethyl sulfate is used to manufacture organic chemicals including pesticides, dyes, drugs, perfumes, fabric softeners, and adhesives [91]. Dimethyl sulfate was also formerly used as a chemical weapon. Occupational exposure to this compound has been linked to cancers of the eye, bronchus, and lung.

Benzene is a known human carcinogen found in gasoline, pesticides, cigarette smoke, and industrial emissions and is a common contaminant detected in food and water [91]. According to the US Department of Health and Human Services, half of the national exposure to benzene comes from cigarette smoke, a known fragile-site-enhancing agent. The optimal concentrations of benzene (500 *μ*g/mL) to induce fragile sites are relevant. Smoke from a smoldering cigarette yields 345–653 *μ*g of benzene, and the average exposure for one hour of driving or riding in a car is about 40 *μ*g benzene (even greater in highly congested areas) [91]. Therefore, the general population is exposed to a level of benzene comparable to the amount able to induce fragile sites, especially under long-term exposure. 

Exposure to benzene due to occupation or geographic location has been linked to leukemia. Recently, Pellegriti et al. observed a significantly higher prevalence of PTC in the Sicilian province of Catania, located near the Mount Etna volcano, compared to other provinces, which could not be explained by industrial pollution or mild iodine deficiencies [92]. The authors suggest that exposure to environmental factors associated with the Mount Etna volcano may be responsible. Benzene can form following the incomplete combustion of organic materials in volcanoes and forest fires [93] and has been detected in lava gases emitted from Mount Etna [94], suggesting fragile site breakage due to benzene exposure may contribute to the increasing incidence of PTC in Catania. Furthermore, we have observed that treatment of HTori-3 cells with levels of benzene previously shown to induce fragile sites [75] results in a statistically significant increase in DNA breakage within *RET* intron 11, and this breakage was specific to fragile sites (unpublished observations).

Diethylnitrosamine (DEN) is found in pesticides, cigarette smoke, industrial pollution, drinking water, and foods and beverages [91]. DEN was previously used as a gasoline and lubricant additive, antioxidant, stabilizer in plastics, and in other manufacturing processes. Although there are no studies relating exposure to DEN with cancer susceptibility in humans, many studies in laboratory animals have shown that DEN exposure can result in the development of various tumors [91]. As with benzene, we found that DNA breakage within *RET* intron 11 was significantly increased in HTori-3 cells following exposure to dosages of DEN previously shown to induce fragile sites [75], and this breakage was specific to fragile sites (unpublished observations).

Atenolol is a common *β*-blocker used to treat hypertension, and peripheral blood lymphocytes from hypertensive patients taking this drug exhibit higher levels of chromatid and chromosomal breaks than normal individuals not taking atenolol; these breaks were preferentially located at fragile sites [74]. Furthermore, blood lymphocytes from patients taking atenolol have significantly more micronuclei than normal patients [95]. Although antihypertensive drugs have been evaluated for carcinogenic effects, studies performed to date may not be complete enough to rule out these drugs as potential cancer-causing agents [96]. Due to the prevalent usage of hypertensive drugs and the ability of atenolol to induce fragile site breakage, usage of such drugs may be an additional risk factor for cancer development, and extensive investigations are needed.

### 4.2. Chemotherapeutic Agents

Several chemotherapeutic agents are known to induce fragile site breakage including actinomycin D, bleomycin, busulfan, camptothecin, chlorambucil, cytosine arabinoside (cytarabine), 5-azacytidine, floxuridine, and methotrexate (Table 1) [75, 86]. The fragile site breakage observed following treatment with these chemotherapeutic agents was multiplied 3- to 8-fold by the addition of caffeine. These chemotherapeutics are commonly used to treat cancer, including leukemias and lymphomas (Table 1). Aside from killing cancer cells, residual doses of chemotherapy drugs can also lead to mutations in healthy cells that could result in a therapy-related second primary tumor. The plasma concentrations of cytarabine derived from the treatment dosage are comparable (~10 *μ*M) or higher (depending on the regimens) [97], to the amount that causes fragile site induction (10 *μ*M).

The rate of second primary cancers is on the rise, and they now account for one in six of all newly diagnosed cancers in the United States [98]. Thyroid cancer has been observed as a secondary cancer following treatment for various cancers, including Hodgkin's lymphoma [99, 100]. Patients with testicular cancer treated with chemotherapy and/or radiation also have a significantly elevated risk for developing thyroid cancer [101]. PTC has been observed in patients treated for osteosarcoma [57, 102–106], including children treated with chemotherapeutic agents (some of which are known to induce fragile sites, including bleomycin, actinomycin D, and methotrexate), but not radiation [106]. PTC was documented as a secondary malignancy following treatment of a pediatric rhabdomyosarcoma patient with only chemotherapeutic drugs, including actinomycin D [107]. PTC has also been observed as a second primary cancer in children treated with chemotherapy alone for acute lymphoblastic leukemia, neuroblastoma, and Ewing's sarcoma [108–111].

Many chemotherapeutic agents target DNA topoisomerases, acting as enzymatic poisons and resulting in an accumulation of double-strand DNA breaks in cells. DNA topoisomerase I activity is vital for common fragile site breakage [112, 113]. Camptothecin, one of the fragile-site-inducing chemotherapeutic agents, is a DNA topoisomerase I poison. The remaining fragile-site-inducing chemotherapeutic agents perturb DNA replication and/or RNA transcription in cells, the mode by which many fragile-site-inducing chemicals lead to chromosomal breakage. Besides the chemotherapeutic drugs already shown to induce fragile sites, many others work in a similar manner by inhibiting DNA topoisomerases and perturbing DNA replication or RNA transcription, suggesting additional drugs may have the ability to induce fragile site breakage.

Together, chemotherapeutic, dietary, and environmental agents represent a diverse spectrum by which individuals can be exposed to and increase their risk of fragile site breakage. Long-term exposure or exposure to significant doses of any of these agents, or a combination of different agents, can increase a person's susceptibility to cancer development, including PTC.

## 5. 5. PTC in Women

**Figure 3:**
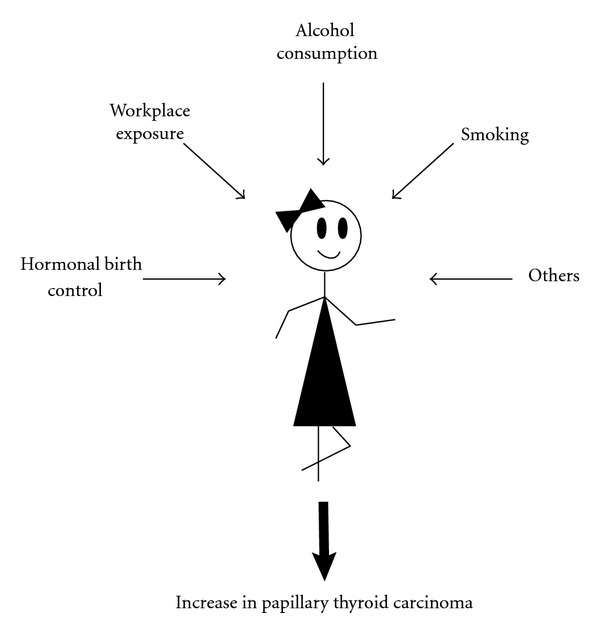
External fragile-site-inducing/enhancing agents as potential risk factors for increased PTC susceptibility in women.

The American Cancer Society 2012 report indicated that the incidence of thyroid cancer among women is three times higher than men [4]. Thyroid cancer is the fastest-growing cancer and the sixth most common among women. Furthermore, the risk of thyroid cancer peaks earlier for women, where most women are diagnosed in the fourth and fifth decades of life compared to the sixth and seventh for men. The rate of PTC among women in the United States tripled between the early 1980s to the mid-2000s, accounting for over 75% of all thyroid cancers diagnosed during this time [3]. Despite this dramatic increase and strong disparity between women and men, there has been no explanation for these observations. However, various fragile-site-inducing factors may contribute to the difference in PTC incidence in women versus men (Figure 3).

One possible explanation for this gender disparity is hormonal differences between men and women. The numbers of chromosomal breaks and sister chromatid exchanges are elevated in women who are pregnant or taking oral contraceptives [114–117]. Furthermore, women currently taking oral contraceptives have an increased incidence of thyroid cancer, including a stronger association with PTC than other subtypes [118]. Variation in the frequency of APH-induced common fragile site breakage was also observed in women during different times of the menstrual cycle, with a significant increase during the luteal phase when progesterone and estradiol levels are at their highest [119]. Estrogen and progesterone levels fluctuate throughout the menstrual cycle, but hormonal birth control prevents ovulation by maintaining consistent hormone levels using synthetic estrogens and progestins. Since the development of oral hormonal contraceptives in 1960, the rate of usage has increased, such that over 10 million women in the United States ages 15 to 44 years currently take oral contraceptives [120]. Further, 82% of reproductive-age women have taken oral hormonal contraceptive pills at some point in their life. The increasing rate of oral contraceptive use among teenage girls ages 13 to 18 is particularly striking [121]. The percentage of women who have ever used high-dose oral contraceptives (emergency contraception) also rose from 4% to 10% from 2002 to 2008 [120]. The emergency contraceptive pill Plan B One-step, which contains 1.5 mg of levonorgestrel, a synthetic form of progesterone, has approximately 10-fold higher levels of the hormone than traditional contraceptives. These data, combined with the previous studies that hormone levels affect breakage within fragile sites, support a possible link between hormonal birth control use and increased fragile site breakage, but more work is needed to prove a causative role.

An increasing presence of women in the workforce may also contribute to elevated incidence of PTC in women. In 1964, only 19 million women in the United States held jobs outside of the home, compared to 65 million women in 2010 [122]. During the 1970s and 1980s, employment in the utility, trade, and transportation industries was most popular among women, and currently these industries are ranked second. Working outside of the home, including commuting to and from work, increases a woman's exposure to environmental mutagens such as benzene and diethylnitrosamine, fragile-site-inducing chemicals found in gasoline fumes and industrial emissions. Furthermore, exposure to the same amount of benzene results in approximately 20% higher levels of benzene metabolites in women versus men [123], indicating an increased susceptibility to fragile site breakage in women. 

In a study examining cancer incidence in US Air Force active duty personnel between 1989 and 2002, thyroid cancer was the third most frequent invasive cancer in women and four times more prevalent than in the general population [124]. Of even greater importance, overall cancer rates among U.S. Air Force personnel were significantly reduced relative to the general population, suggesting occupational exposure may contribute to the difference in thyroid cancer rates. Active duty Air Force personnel encounter unique occupational exposures that can induce fragile site breakage, including jet fuel, high altitudes, and chemical weapons.

Trends in cigarette smoking and alcohol consumption may also contribute to increased PTC among women. Cigarette smoking has been declining in the United States over the past several decades, but the rate of decrease varies between men and women. The number of adult men that smoke decreased by 11.1% between 1965 to 2009, but the number of women only decreased by 0.5% [125]. A high level of alcohol consumption among young women is also very striking. Nearly 66% of women aged 18–44 years consume alcohol, and 14% binge drink [126]. In 2009, approximately 75% of high school girls reported having drank alcohol and nearly half currently consumed alcohol, both higher proportions than in their male counterparts [127]. Also, between 1979 and 2003 binge drinking among women ages 21–23 dramatically increased—40% among college students and 20% among nonstudents—compared to a 10% decrease among men [128]. Alcohol metabolism also differs between men and women, such that when drinking the same quantity of alcohol as men, women have a higher blood alcohol content, which enhances the potential of fragile site breakage.

Due to the significant increase in PTC diagnoses among women in the past several decades, especially among younger women, it is essential to investigate the mechanisms behind this trend. Several factors—such as hormone levels, increased exposure to environmental agents at the workplace, and behaviors like cigarette usage and alcohol consumption—suggest fragile site breakage may contribute to some of the increased incidence of PTC among women. However, more work is needed to clarify these risk factors.

## 6. Conclusion

The dramatic increase of thyroid cancer in the past several decades is alarming, and it is the fastest-growing cancer among women. A variety of potential risk factors for thyroid cancer, including radiation exposure, improved detection techniques, and obesity, have been proposed. Herein we provide evidence for an additional risk factor, chromosomal fragile site breakage. Chromosomal fragile sites are stable under normal conditions, but DNA breakage at these sites can be induced through exposure to many external agents. The genes involved in *RET/PTC1* rearrangements, *RET* and *CCDC6*, are located within common fragile sites, and direct evidence shows that induction of fragile sites results in chromosomal breakage within these genes and ultimately leads to the formation of *RET/PTC1* rearrangements. *RET/PTC1* rearrangements are commonly seen in PTC tumors and are especially represented in case of sporadic PTC. Therefore, exposure to external fragile-site-inducing conditions could account for some sporadic PTC tumors and may contribute to the increase in PTC incidence.

Various external agents can induce fragile site breakage, including dietary, environmental, and chemotherapeutic compounds. Furthermore, women are especially susceptible to many of these agents, and trends over the past several decades support the involvement of fragile sites in the increased incidence of PTC in women. Therefore exposure to external fragile-site-inducing agents, along with genetic factors, may predispose an individual to PTC development, making fragile sites an additional potential risk factor for thyroid cancer. Further work is needed to elucidate the impact of fragile-site-inducing agents in the formation of PTC, such that the treatment of patients can be tailored and further growth in thyroid cancer incidence can be reduced.

## Figures and Tables

**Table 1:** External Fragile Site-Inducing/Enhancing Agents.

Agents	Applications	References
Dietary and environmental		
Atenolol	Hypertension drug	[74]
Benzene	Cigarette smoke, gasoline, pesticides, food, water	[75]
Caffeine	Dietary agent	[76, 77]
Carbon tetrachloride	Refrigerants, pesticides	[75]
Cigarette smoke	Dietary and environmental agent	[78, 79]
Diethylnitrosamine	Cigarette smoke, pesticides, food, beverage	[75]
Dimethyl sulfate	Dyes, drugs, perfumes, pesticides	[75]
Ethanol	Dietary agent	[80, 81]
Hypoxia	Low oxygen, tumor microenvironment	[82]
Pesticides	Dietary and environmental agent	[83–85]

Chemotherapeutics		
5-Azacytidine	Myelodysplastic syndrome, leukemia	[75]
Actinomycin D	Sarcoma, Wilms' tumor, germ cell, testicular, Melanoma, neuroblastoma, retinoblastoma	[75]
Bleomycin	Squamous cell, melanoma, sarcoma, testicular, Hodgkin's and non-Hodgkin's lymphoma	[75]
Busulfan	Chronic myelogenous leukemia	[75]
Camptothecin	Colon, rectal	[86]
Chlorambucil	Chronic lymphocytic leukemia, Hodgkin's and non-Hodgkin's lymphoma, breast, ovarian, testicular	[75]
Cytosine arabinoside	Leukemia, lymphoma	[75]
Floxuridine	Colon, kidney, stomach	[75]
Methotrexate	Breast, head and neck, lung, stomach, esophageal, sarcoma, non-Hodgkin's lymphoma, acute lymphoblastic leukemia	[75]
